# Income and patient-reported outcomes (PROs) after primary total knee arthroplasty

**DOI:** 10.1186/1741-7015-11-62

**Published:** 2013-03-06

**Authors:** Jasvinder A Singh, David G Lewallen

**Affiliations:** 1 Medicine Service and Center for Surgical Medical Acute are Research and Transitions (C-SMART), Birmingham VA Medical Center, 700 19th Street South Birmingham, AL 35233, USA; 2 Department of Medicine at School of Medicine, and Division of Epidemiology at School of Public Health, University of Alabama, 20th Street South, FOT 805B, Birmingham, AL 35294, USA; 3 Department of Orthopedic Surgery, Mayo Clinic College of Medicine, 200 1st St SW, Rochester, MN 55905, USA

**Keywords:** arthroplasty, income, joint replacement, patient-reported outcomes, risk factor, total knee replacement

## Abstract

**Background:**

To assess whether income is associated with patient-reported outcomes (PROs) after primary total knee arthroplasty (TKA).

**Methods:**

We used prospectively collected data from the Mayo Clinic Total Joint Registry to assess the association of income with index knee functional improvement, moderate to severe pain and moderate to severe activity limitation at 2-year and 5-year follow-up after primary TKA using multivariable-adjusted logistic regression analyses.

**Results:**

There were 7, 139 primary TKAs at 2 years and 4, 234 at 5 years. In multivariable-adjusted analyses, at 2-year follow-up, compared to income > US$45, 000, lower incomes of ≤ US$35, 000 and > US$35, 000 to 45, 000 were associated (1) significantly with moderate to severe pain with an odds ratio (OR) 0.61 (95% CI 0.40 to 0.94) (*P *= 0.02) and 0.68 (95% CI 0.49 to 0.94) (*P *= 0.02); and (2) trended towards significance for moderate to severe activity limitation with OR 0.78 (95% CI 0.60 to 1.02) (*P *= 0.07) and no significant association with OR 0.96 (95% CI 0.78 to 1.20) (*P *= 0.75), respectively. At 5 years, odds were not statistically significantly different by income, although numerically they favored lower income. In multivariable-adjusted analyses, overall improvement in knee function was rated as 'better' slightly more often at 2 years by patients with income in the ≤ US$35, 000 range compared to patients with income > US$45, 000, with an OR 1.9 (95% CI 1.0 to 3.6) (*P *= 0.06).

**Conclusions:**

We found that patients with lower income had better pain outcomes compared to patients with higher income. There was more improvement in knee function, and a trend towards less overall activity limitation after primary TKA in lower income patients compared to those with higher incomes. Insights into mediators of these relationships need to be investigated to understand how income influences outcomes after TKA.

## Background

Total knee arthroplasty (TKA), a common surgical procedure with a rapidly increasing annual volume in the USA [1], is associated with significant improvement in pain, function and quality of life [2]. However, 10% to 30% of patients continue to have refractory pain and/or significant functional limitation even years after TKA or total hip arthroplasty [3-5]. Identification of significant predictors of post-arthroplasty pain and functional limitation [6-9] will allow us to design interventions targeting risk modifiable factors, with a likelihood of improving outcomes. Much emphasis in the previous studies has been placed on implant and surgical aspects of arthroplasty and its demographic predictors, with very few studies focusing on socioeconomic predictors. It is well established that patients with low income are less likely to receive total joint replacement [10-14], which also partially explains the racial disparity in total joint replacement utilization [12,13,15,16]. Need and willingness to undergo joint arthroplasty does not differ by income level [17].

Few studies have examined the association of lower income with arthroplasty outcomes. To the best of our knowledge, there has been only one study in a TKA cohort [18]. This study found no differences in pain and function outcomes 2 years after TKA by income level, but higher gains in a lower income group [18]. Additional studies in hip arthroplasty populations have yielded contradictory results. Lower income was either associated with higher risk of medical complications in a population-based Italian study [14] and in the Medicare population [19] or not associated with patient satisfaction or likelihood of achieving excellent hip function in a US study [20] in hip arthroplasty cohorts. Since data are based on small samples with limited follow-up and not conclusive, well designed studies with adequate power that assess this relationship in larger samples post TKA are needed. We hypothesized that lower income would predict poorer absolute scores in pain and function post TKA, but more improvement in function outcomes with TKA compared to preoperative scores. Using data from a large institutional total joint registry, we examined the association of income with moderate to severe pain, moderate to severe activity limitation and improvement in knee function at 2 and 5 years after primary TKA.

## Methods

The current study used the data collected in the Mayo Total Joint Registry, one of the largest US joint registries [21]. The Mayo Total Joint Registry collects prospective data on all joint replacements performed at the Mayo Clinic including patient demographics and clinical characteristics (body mass index (BMI)); reoperations, complications and whether the implant was in place or removed; current radiographs; and pain and function assessments [22,23]. The Mayo Knee questionnaire [24] is reliable and is based on the American Knee Society scale, a validated instrument that is most commonly used in evaluation of knee arthroplasty patients [25-27]. The pain and function questions analyzed in this study from the Mayo Knee questionnaire are the same as those in the Knee Society Score [28]. Questionnaires are administered to all patients undergoing knee arthroplasty at the Mayo Clinic, and these data have been captured electronically since 1993. The questionnaires are mailed to the patients, administered during the clinic visit or by telephone by experienced, dedicated joint registry staff at 2-year and 5-year timepoints after TKA. Patients were included in this study if they had undergone a primary TKA during 1993 to 2005 and had responded to either a 2-year or 5-year follow-up questionnaire. The study was approved by the Mayo Clinic's Institutional Review Board.

### Predictor variable and its definition

The predictor of interest was the patient's income at the time of index arthroplasty, as assessed based on patient zip codes and the median household income for geographical area using the census data for the respective year of survey. Income was categorized into ≤ US$35, 000, > US$35, 000 to US$45, 000 and > US$45, 000 levels, as previously [8,9,29,30]. Categories were created based on the fact that US$35, 000 was twice the poverty level income for a family of four in 1998 to 1999, the midpoint of our study [31]. Additional sensitivity analyses were performed with a different income cut-off.

### Outcomes of interest

We assessed three key patient-reported outcomes at 2 years and 5 years after TKA. Two outcomes indicated 'state' post TKA (moderate to severe pain and moderate to severe activity limitation) and one indicated 'change' after TKA (knee function improvement from before to after surgery):

(1) Moderate to severe knee pain: assessed based on the responses to a question regarding pain in knee similar to the pain question in the Knee Society Scale [25] 'Do you have pain in the knee in which the joint was replaced?', with responses of no pain, mild (occasional), stairs only, walking and stairs combined into reference category and moderate (occasional), moderate (continuous) and severe pain combined into moderate to severe pain, similar to previous studies [5,9,32].

(2) Moderate to severe activity limitation: this was defined as the presence of moderate or severe limitation in two or more of the three activities queried (walking, stairs, rising chair), as previously [29,32].

(3) Improvement in knee function: this was assessed with the single question 'Compared to your condition before your knee surgery, how would you rate your knee function?', with responses of much better, better, same or worse. Responses of same and worse were combined into the reference category, as described previously [32].

### Covariates of interest

The covariates included factors previously shown to be associated or potentially associated with pain and function outcomes after TKA [5,9,23,33-35]. These included patient characteristics (age, gender, BMI), comorbidity, American Society of Anesthesiologists (ASA) score, preoperative pain and preoperative activity limitation (in respective models), implant fixation (cemented/hybrid versus not cemented), underlying diagnosis (osteoarthritis, rheumatoid/inflammatory arthritis or other) and distance from medical center (categorized < 100, 100 to 500 and > 500 miles/overseas). Age was categorized into ≤ 60, 61 to 70, 71 to 80 and > 80 categories, as previously [5,36]. BMI was categorized, as previously [37], into ≤ 25, 25.1 to 29.9, 30 to 34.9, 35 to 39.9 and ≥ 40 kg/m^2^. Comorbidity was measured by Deyo-Charlson score, the most commonly used validated comorbidity measure [38] consisting of a weighted scale of 19 comorbidities (including cardiac, pulmonary, renal, hepatic disease, diabetes, cancer, HIV and so on), expressed as a summative score [39,40], with a higher score indicates more comorbidity. ASA score, a validated measure of perioperative and postoperative outcomes, was categorized as class I to II versus III to IV [41,42].

### Statistical analyses

Responder and non-responder characteristics were compared using logistic regression analyses. Multivariable-adjusted logistic regression analyses were performed separately for each of the three prespecified outcomes at prespecified timepoints of 2 and 5 years (six models). For these analyses, we used a generalized estimating equations (GEE) approach to adjust for the correlation between observations on the same subject (for example, if a patient underwent right and left primary TKA). The main multivariable-adjusted analyses adjusted for several baseline variables including age category, gender, BMI category, comorbidity, ASA class, operative diagnosis, distance from the medical center and implant fixation. Since improvement in knee function had three categories (much better, better versus same/worse (reference)), we used polytomous logistic regression, which does not make any assumption of parallel slopes (as is made in ordinal logistic regression) [43,44]. Odds ratios (OR) and the 95% confidence intervals (CI) are presented. A *P *value < 0.05 was considered significant. We performed additional sensitivity analyses for pain and activity limitation outcomes: (1) adding preoperative pain to the function outcome analyses and preoperative function to the pain analyses; (2) varying the income category cut-offs to two times the poverty level in 2005 (US$39, 000); and (3) including age and BMI as continuous variables, instead of categorical variables. Since improvement in knee function incorporates preoperative status, these models were not additionally adjusted for preoperative variables, to avoid overadjustment.

## Results

### Patient characteristics and responder bias

Patient characteristics for TKA cohorts at 2-year and 5-year follow-up are shown in Table 1. At 2-year follow-up, the mean age was 68 years, 56% were women, 9% had BMI ≥ 40 kg/m^2^, and 42% had ASA class III or IV. Medical comorbidities, anxiety and depression were common. Detailed characteristics for 5-year follow-up are provided in Table 1.

**Table 1:** Clinical and demographic characteristics of patients

	Primary TKA, n^a ^(%) unless specified otherwise	
	
	2 years (n = 7, 139)	5 years, (n = 4, 234)
Men/women	3, 162 (44.3%)/3, 977 (55.7%)	1903 (44.9%)/2, 331 (55.1%)
Age, mean ± SD	68.4 ± 10.0	68.4 ± 9.6
Age groups, n (%):		
≤ 60 years	1, 313 (18.4%)	745 (17.6%)
> 60 to 70 years	2, 531 (35.5%)	1, 576 (37.2%)
> 70 to 80 years	2, 734 (38.3%)	1, 617 (38.2%)
> 80 years	561 (7.9%)	296 (7.0%)
Body mass index, kg/m^2^:		
< 25	934 (13.1%)	566 (13.4)%
25 to 29.9	2, 479 (34.7%)	1, 525 (36.0%)
30 to 34.9	2, 092 (29.3%)	1, 250 (29.5%)
35 to 39.9	976 (13.7%)	573 (13.5%)
≥ 40	605 (8.5%)	303 (7.2%)
ASA score:		
Class I to II	4, 115 (57.6%)	2, 467 (58.3%)
Class III to IV	3, 006 (42.1%)	1, 741 (41.1%)
Deyo-Charlson Index, mean ± SD	1.2 ± 1.9	1.1 ± 1.9
Key Deyo-Charlson comorbidities:		
Myocardial infarction	341 (4.8%)	202 (4.8%)
Peripheral vascular disease	370 (5.2%)	190 (4.5%)
Renal disease	397 (5.6%)	175 (4.1%)
Chronic obstructive pulmonary disease	762 (10.7%)	408 (9.6%)
Diabetes	669 (9.4%)	349 (8.2%)
Connective tissue disease	520 (7.3%)	352 (8.3%)
Anxiety	465 (6.5%)	220 (5.2%)
Depression	741 (10.4%)	334 (7.9%)
Distance from medical center:		
0 to 100 miles	3, 669 (51.4%)	2, 080 (49.1%)
> 100 to 500 miles	2, 731 (38.3%)	1, 653 (39.0%)
> 500 miles	541 (7.6%)	327 (7.7%)
Diagnosis:		
Inflammatory arthritis	256 (3.6%)	189 (4.5%)
Osteoarthritis	6, 710 (94.0%)	3, 922 (92.6%)
Other	172 (2.4%)	123 (2.9%)

^a^Missing values for 2-year cohort: 27 were missing body mass index (BMI), 18 were missing American Society of Anesthesiologists (ASA) class, 3 were missing Charlson Index scores, 198 were missing the distance variable, 1 was missing operative diagnosis. Missing values for 5-year cohort: 17 were missing BMI, 26 were missing ASA class, 174 were missing the distance variable, 23 were missing operative diagnosis.

The response rate was 65% (7, 139/10, 957) at 2 years and 57% (4, 234/7, 404) at 5 years. For primary TKA 2-year and 5-year follow-up, male gender, osteoarthritis diagnosis, older age, lower ASA class, lower Deyo-Charlson comorbidity index score and shorter distance from medical center (< 100 miles) were associated with greater odds of response.

Unadjusted prevalence of suboptimal pain, activity limitation and index knee function improvement outcomes is shown in Table 2.

**Table 2:** Prevalence of pain and function outcomes by income category

	Moderate to severe pain	Moderate to severe functional limitation	Overall knee status	
			
			Better	Much better
2 years:				
≤ US$35, 000	5.7%	19.6%	10.9%	85.7%
> US$35, 000 to US$45, 000	6.7%	22.9%	8.8%	87.5%
> US$45, 000	8.2%	23.1%	8.5%	87.6%
5 years:				
≤ US$35, 000	7.2%	28.0%	9.4%	85.6%
> US$35, 000 to US$45, 000	7.5%	29.7%	9.3%	85.4%
> US$45, 000	8.2%	29.6%	9.7%	84.4%

### Income and outcomes after primary TKA

#### State outcomes

For unadjusted models, the lowest income category (≤ US$35, 000) was associated with significantly lower odds of moderate to severe pain (*P *= 0.01) and moderate to severe activity limitation at 2 years (*P *= 0.04) and borderline significant association of the US$35, 000 to 45, 000 income category with lower odds of moderate to severe pain at 2 years, compared with the highest income category (*P *= 0.06; Table 3). In multivariable-adjusted models that also adjusted for the respective preoperative variable, both lower income categories (≤ US$35, 000 and > US$35, 000 to US$45, 000) were associated with significantly lower odds of moderate severe pain (*P *= 0.02 each), and a borderline significant difference in odds of moderate to severe activity limitation at 2 years post primary TKA, compared to the higher income group (> US$45, 000; *P *= 0.07) (Table 4). Similar differences were seen at 5 years, but these were not significant. Sensitivity analyses that adjusted for preoperative pain in the activity limitation model (and vice versa; see Additional file 1), varied the income categories (Additional file 2) or examined age and BMI as continuous variables (Additional file 3), did not effect the interpretation of study results for moderate to severe pain or moderate to severe activity limitation outcomes.

**Table 3:** Unadjusted association of income with pain, activity limitation and improvement in knee function after primary total knee arthroplasty

	2 years				5 years			
	
	Odds ratio (95% CI)	*P *value	Odds ratio (95% CI)	*P *value	Odds ratio (95% CI)	*P *value	Odds ratio (95% CI)	*P *value
Pain/function:	Moderate to severe pain		Moderate to severe functional limitation		Moderate to severe pain		Moderate to severe functional limitation	
≤ US$35, 000	0.68 (0.50 to 0.92)	0.01	0.82 (0.67 to 0.99)	0.04	0.86 (0.60 to 1.22)	0.39	0.92 (0.74 to 1.15)	0.47
> US$35, 000 to US$45, 000	0.80 (0.64 to 1.01)	0.06	0.99 (0.85 to 1.15)	0.90	0.90 (0.65 to 1.25)	0.55	1.00 (0.82 to 1.23)	0.99
> US$45, 000 (ref)	1.00		1.00		1.00		1.00	
Improvement:	Better		Much better		Better		Much better	
≤ US$35, 000	1.5 (0.9 to 2.4)	0.13	1.1 (0.7, 1.7)	0.61	1.2 (0.7 to 2.0)	0.57	1.2 (0.8 to 1.9)	0.39
> US$35, 000 to US$45, 000	1.1 (0.7 to 1.6)	0.72	1.0 (0.7 to 1.5)	0.83	1.1 (0.7 to 1.7)	0.78	1.1 (0.8, 1.7)	0.56
> US$45, 000 (ref)	1.00		1.00		1.00		1.00	

**Table 4:** Multivariable-adjusted association of income with pain and activity limitation after primary total knee arthroplasty

	2 years				5 years			
	
	Moderate to severe pain		Moderate to severe functional limitation		Moderate to severe pain		Moderate to severe functional limitation	
	
	Odds ratio (95% CI)	*P *value	Odds ratio (95% CI)	*P *value	Odds ratio (95% CI)	*P *value	Odds ratio (95% CI)	*P *value
≤ US$35, 000	0.61 (0.40 to 0.94)	0.02	0.78 (0.60 to 1.02)	0.07	0.77 (0.50 to 1.19)	0.24	0.91 (0.68 to 1.25)	0.56
> US$35, 000 to US$45, 000	0.68 (0.49 to 0.94)	0.02	0.96 (0.78 to 1.20)	0.75	0.90 (0.60 to 1.34)	0.60	1.09 (0.83 to 1.44)	0.52
> US$45, 000 (ref)	1.00		1.00		1.00		1.00	

Data were additionally adjusted for the following variables at baseline: age category, gender, body mass index (BMI) category, Deyo-Charlson index, American Society of Anesthesiologists (ASA) class, distance, operative diagnosis, implant fixation, preoperative pain (for pain models) and preoperative functional limitation (for functional limitation models). Significant variables in each model included the following: (1) 2-year pain model: age (*P *= 0.03) and preoperative pain (*P *= 0.003); (2) 2-year activity limitation model: age (*P *< 0.001), gender (*P *< 0.001), body mass index (*P *< 0.001), ASA class (*P *< 0.001) and preoperative activity limitation (*P *< 0.001); (3) 5-year pain model: ASA class (*P *< 0.001), distance from medical center (*P *= 0.02) and implant fixation (*P *= 0.01); (4) 5-year activity limitation model: age (*P *< 0.001), gender (*P *< 0.001), body mass index (*P *< 0.001), ASA class (*P *< 0.001), Deyo-Charlson index (*P *= 0.02) and preoperative activity limitation (*P *< 0.001).

#### Change outcome

For unadjusted models, the lowest income category (≤ US$35, 000) was associated with higher odds of 1.5 for better index knee function at 2 years, but this did not reach significance (*P *= 0.13; Table 3). In multivariable-adjusted models, a lower income (≤ US$35, 000) was associated with higher odds of having more index knee function improvement ('better') relative to the preoperative function, as compared to the higher income category (> US$45, 000) (*P *= 0.06; Table 5). Numerically higher but statistically non-significant odds of 'much better' functional improvement were seen at 2 and 5 years in those with a lower income (≤ US$35, 000). Less impressive differences were seen in the > US$35, 000 to US$45, 000 income category.

**Table 5:** Multivariable-adjusted association of income with improvement in knee function after primary TKA

	2 years				5 years			
	
	Better		Much better		Better		Much better	
	
	Odds ratio (95% CI)	*P *value	Odds ratio (95% CI)	*P *value	Odds ratio (95% CI)	*P *value	Odds ratio (95% CI)	*P *value
≤ US$35, 000	1.9 (1.0 to 3.6)	0.06	1.4 (0.8 to 2.5)	0.26	1.7 (0.8 to 3.4)	0.16	1.5 (0.9 to 2.8)	0.15
> US$35, 000 to US$45, 000	1.4 (0.8 to 2.4)	0.25	1.4 (0.9 to 2.3)	0.14	1.2 (0.6 to 2.2)	0.64	1.0 (0.6 to 1.7)	0.93
> US$45, 000 (ref)	1.00		1.00		1.00		1.00	

Data additionally adjusted for the following variables at baseline: age category, gender, body mas index (BMI) category, Deyo-Charlson index, American Society of Anesthesiologists (ASA) class, distance, operative diagnosis, implant fixation, preoperative pain (for pain models) and preoperative functional limitation (for functional limitation models). Significant variables in each model included the following: (1) 2-year model: ASA class III/IV (*P *< 0.01) with lower odds for much better status and distance > 100 to 500 miles with lower odds for much better status (*P *= 0.03); and preoperative activity limitation (*P *< 0.001); (2) 5-year model: ASA class III/IV (*P *< 0.01) with lower odds for much better status.

## Discussion

In this study, interestingly we found that lower income was associated with better pain outcome at 2 years after primary TKA. We also found that patients with lower income saw more improvement in overall knee function from pre-TKA to post-TKA evaluation, compared to higher income patients. Similar, but non-significant, associations were noted at 5 years. Several study findings merit further discussion, but need to be interpreted considering study limitations.

Our study has several limitations. A non-response bias may limit the generalizability of our findings. The response rate of 65% and 57% for pain and function surveys at 2 and 5 years is similar to the average survey response rate of 60% for large surveys such as ours [45]. However, the study cohort's clinical and demographic characteristics are similar to TKA cohorts reported previously. Income was based on zip code median income, and not actual personal income, which may have led to misclassification bias. Whether this biased our results towards or away from the null hypothesis is not entirely clear. People with the same income in different parts of the US (New York versus Midwest versus South) have slightly different purchasing power. Although this does not impact the observed associations, the variation in purchasing power by geography may impact access to health care even for those with similar socioeconomic status. Even though we specified the three outcomes *a priori*, with the Bonferroni adjustment, our findings may be considered borderline significant for such a conservative approach (with a corrected *P *value of 0.017 for three comparisons). Study strengths included adjustment for important covariates, robust results in multivariable-adjusted analyses and multiple sensitivity analyses, a large sample size with lower risk of a type II error, analyses of both pain and function outcomes and examination of both 'state' and 'change' variables for outcomes.

A key interesting finding in our study was the lower risk of moderate or severe pain post TKA in patients with lower income. In a recent TKA study that included patients from four countries including the US, lower income was reported as not significantly associated with pain and function outcomes up to 2 years after TKA (*P *= 0.07) [18]. On closer inspection, the Western Ontario and McMaster Universities Arthritis Index (WOMAC) pain scores (higher = worse) for income categories < US$15, 000, US$15, 000 to 30, 000, US$30, 000 to 45, 000, US$45, 000 to 60, 000 and > US$60, 000 were 78, 83, 80, 82 and 87 respectively (*P *= 0.014); that is, significantly less pain in patients with lower income at 1 year [18]. In fact similar lower scores were also noted for lower income categories at 2 years, but the level of statistical significance was not met (*P *= 0.07), likely due to a small sample size. Thus, this earlier study did provide hypothesis generation for better pain outcomes in lower income groups, now confirmed in our study with ten times the sample size. Most literature regarding poorer health outcomes in those with lower income comes from pregnancy and other conditions. However, several important considerations make the findings from our study and the earlier international randomized study compatible, rather than contradictory, with the existing literature on income and outcomes.

First, the outcome described here is a patient-reported outcome (pain), which is likely influenced as much or more by social contextual factors as it is impacted by comorbidity load (presumed higher in lower income individuals). This is in contrast to mortality and other end-organ damage outcomes (the so called 'hard outcomes'), which may be influenced far more by comorbidity. Second, lower income [46] and poorer preoperative functional status [47] have both been shown to be associated with higher expectations of joint replacement. Higher expectations are associated with better outcomes [48,49]. Thus, lower income individuals would be expected to report better patient-reported outcomes after TKA than those with higher incomes. Third, studies in other diseases indicated that several social contextual factors including social support impact health outcomes, perhaps more than income [50-52]. More studies are needed to understand the association of income and other contextual factors with pain and function outcomes after TKA.

Another interesting finding from our study was that patients in the lowest income category were twice as likely than those in the highest income category to report a 'better' improvement in the index knee function 2 years after primary TKA. This finding should not be surprising at all considering that those in the lower income categories have worse preoperative functional status [18,20,53], but similar postoperative function after TKA [18,53], than those in the higher categories. Since those with a lower income have worse scores preoperatively, they have a much greater chance to improve their knee function, compared to those on a higher income. Our study corroborates and extends these earlier findings. The simple knee function improvement question used in this study has been used previously [32], and may be easily implemented in clinical practice in addition to longer composite pain and function instruments used in clinical research. The knee function improvement used in this study is clinically meaningful at patient level, and much easier to interpret as compared to a change in mean function score, usually presented for an entire cohort (some patients improving dramatically, others not at all). In addition, activity limitations were lesser in lower income categories compared to the highest income category, although they did not reach statistical significance.

## Conclusions

In summary, patients in lower income groups had better pain outcomes and more improvement in index knee function at 2 years after primary TKA. Similar differences by income were noted at 5 years, although they were not significant. These data are reassuring in that, at least for primary TKA, lower income is not a risk factor for poor outcomes; on the contrary it may be associated with better outcomes. Future studies should investigate the reasons for better outcomes after TKA in patients with lower incomes.

## Abbreviations

ASA: American Society of Anesthesiologists; BMI: body mass index; GEE: generalized estimating equations; PROs: patient-reported outcomes; TKA: total knee arthroplasty.

## Competing interests

The authors report no financial conflicts related directly to this study. JAS has received research grants from Takeda and Savient; and consultant fees from URL pharmaceuticals, Savient, Takeda, Regeneron, Allergan, Ardea and Novartis. DGL has received royalties/speaker fees from Zimmer, Orthosonic and Osteotech, has been a paid consultant to and owns stock in Pipeline Biomedical and has received institutional research funds from DePuy, Stryker, Biomet and Zimmer.

The views expressed in this article are those of the authors and do not necessarily reflect the position or policy of the Department of Veterans Affairs or the United States government.

## Authors' contributions

JAS developed the study concept and protocol, obtained IRB approval, designed and reviewed statistical analyses, drafted and revised the manuscript and submitted the manuscript. DGL reviewed the study concept and protocol, and participated in its design and coordination, reviewed analyses and reviewed and revised the manuscript. Both authors read and approved the final manuscript.

## Pre-publication history

The pre-publication history for this paper can be accessed here:

http://www.biomedcentral.com/1741-7015/11/62/prepub

## Supplementary Material

Additional file 1 **Sensitivity analyses additionally adjusting multivariable analyses for preoperative activity limitation in pain outcome models and preoperative pain in activity limitation models**. This table shows the sensitivity analyses that adjusted the main models for pain and activity limitation additionally for preoperative pain and activity limitation, respectively. Click here for file

Additional file 2 **Sensitivity analyses using a different income category cut-off of US$39, 000 for the lowest category in multivariable-adjusted analyses**. This table shows the sensitivity analyses that used a different cut-off for income for the lowest income category of US$39, 000 instead of US$35, 000 (as in the main model). Click here for file

Additional file 3 **Sensitivity analyses using body mass index (BMI) and age as continuous variables in multivariable-adjusted analyses**. this table shows the sensitivity analyses that adjusted the main model for a continuous age and BMI variable instead of the categorical variable for both. Click here for file
