# Supplementary material for: Income and patient-reported outcomes (PROs) after primary total knee arthroplasty
Source: BMC Med. 2013 Mar 6;11:62. doi: 10.1186/1741-7015-11-62 (PMC3641978; doi:10.1186/1741-7015-11-62)
Supplement: Additional file 1 — Sensitivity analyses additionally adjusting multivariable analyses for preoperative activity limitation in pain outcome models and preoperative pain in activity limitation models. This table shows the sensitivity analyses that adjusted the main models for pain and activity limitation additionally for preoperative pain and activity limitation, respectively. [file 1741-7015-11-62-S1.DOCX]

Additional File

Additional File 1. **Sensitivity analyses additionally adjusting multivariable analyses for preoperative activity limitation in pain outcome models and preoperative pain in activity limitation models**

|  | **2-year** | | | | **5-year** | | | |
| --- | --- | --- | --- | --- | --- | --- | --- | --- |
|  | **Moderate-severe pain** | | **Moderate severe functional limitation** | | **Moderate-severe pain** | | **Moderate severe functional limitation** | |
|  | **Odds Ratio (95% CI)** | **p-value** | **Odds Ratio (95% CI)** | **p-value** | **Odds Ratio (95% CI)** | **p-value** | **Odds Ratio (95% CI)** | **p-value** |
| ≤$35K | **0.62**  **(0.40, 0.96)** | **0.03** | 0.79  (0.51, 1.22) | 0.29 | 0.78  (0.50, 1.22) | 0.28 | 0.77  (0.59, 1.01) | 0.06 |
| >$35K-$45K | **0.69**  **(0.50, 0.95)** | **0.02** | 0.82  (0.59, 1.14) | 0.24 | 0.89  (0.59, 1.33) | 0.57 | 0.95  (0.76, 1.19) | 0.57 |
| >$45K (ref) | 1.00 |  | 1.00 |  | 1.00 |  | 1.00 |  |
