# Supplementary material for: Income and patient-reported outcomes (PROs) after primary total knee arthroplasty
Source: BMC Med. 2013 Mar 6;11:62. doi: 10.1186/1741-7015-11-62 (PMC3641978; doi:10.1186/1741-7015-11-62)
Supplement: Additional file 2 — Sensitivity analyses using a different income category cut-off of US$39, 000 for the lowest category in multivariable-adjusted analyses. This table shows the sensitivity analyses that used a different cut-off for income for the lowest income category of US$39, 000 instead of US$35, 000 (as in the main model). [file 1741-7015-11-62-S2.DOCX]

Additional File 2. **Sensitivity analyses using a different income category cut-off*** **in multivariable-adjusted analyses**

|  | **2-year** | | | | **5-year** | | | |
| --- | --- | --- | --- | --- | --- | --- | --- | --- |
|  | **Moderate-severe pain** | | **Moderate severe functional limitation** | | **Moderate-severe pain** | | **Moderate severe functional limitation** | |
|  | **Odds Ratio (95% CI)** | **p-value** | **Odds Ratio (95% CI)** | **p-value** | **Odds Ratio (95% CI)** | **p-value** | **Odds Ratio (95% CI)** | **p-value** |
| ≤$39K* | **0.67**  **(0.48, 0.93)** | **0.02** | 0.85  (0.68, 1.06) | 0.15 | 0.86  (0.59, 1.25) | 0.43 | 1.00  (0.77, 1.31) | 0.99 |
| >$39K-$45K | **0.64**  **(0.43, 0.96)** | **0.03** | 1.01  (0.78, 1.30) | 0.95 | 0.80  (0.48, 1.35) | 0.41 | 1.05  (0.75, 1.48) | 0.76 |
| >$45K (ref) | 1.00 |  | 1.00 |  | 1.00 |  | 1.00 |  |

Income cut off was varied to 2-times the 2005 poverty cut-off of annual income of $19.5K at $39K, instead of the main analyses cut-off of $35K
