# Supplementary material for: Income and patient-reported outcomes (PROs) after primary total knee arthroplasty
Source: BMC Med. 2013 Mar 6;11:62. doi: 10.1186/1741-7015-11-62 (PMC3641978; doi:10.1186/1741-7015-11-62)
Supplement: Additional file 3 — Sensitivity analyses using body mass index (BMI) and age as continuous variables in multivariable-adjusted analyses. this table shows the sensitivity analyses that adjusted the main model for a continuous age and BMI variable instead of the categorical variable for both. [file 1741-7015-11-62-S3.DOCX]

Additional File 3. **Sensitivity analyses using BMI and age as continuous variables in multivariable-adjusted analyses**

|  | **2-year** | | | | **5-year** | | | |
| --- | --- | --- | --- | --- | --- | --- | --- | --- |
|  | **Moderate-severe pain** | | **Moderate severe functional limitation** | | **Moderate-severe pain** | | **Moderate severe functional limitation** | |
|  | **Odds Ratio (95% CI)** | **p-value** | **Odds Ratio (95% CI)** | **p-value** | **Odds Ratio (95% CI)** | **p-value** | **Odds Ratio (95% CI)** | **p-value** |
| ≤$35K | **0.59**  **(0.38, 0.89)** | **0.01** | **0.76**  **(0.58, 0.99)** | **0.04** | 0.76  (0.50, 1.17) | 0.21 | 0.88  (0.65, 1.20) | 0.40 |
| >$35K-$45K | **0.67**  **(0.48, 0.92)** | **0.01** | 0.96  (0.78, 1.20) | 0.74 | 0.90  (0.60, 1.34) | 0.61 | 1.06  (0.80, 1.39) | 0.69 |
| >$45K (ref) | 1.00 |  | 1.00 |  | 1.00 |  | 1.00 |  |
